# Removal of a Nail from the Lungs by Tracheotomy

**Published:** 1853-10

**Authors:** 


*Removal of a Nail from the Lungs by Tracheotomy.*

By PAUL F. EVE, M. D.

From a monograph thus entitled, we extract the following account of a very interesting case. In our own experience, we have but once met with the presence of a foreign body in the lung; and the fatal result of this case, occurring after a long illness from phthisis, produced by the irritation of the foreign body, gives to the present more successful case, an interest which we are confident our readers will share with us:

"On the 20th of June, the Rev. Mr. Lane, residing near Talladega, Alabama, came to Augusta, seeking professional advice for his little son, who, two weeks previously, had placed a fourpenny nail in the hole of a cotton spool with the design of making a whistle, but, unfortunately, in taking a deep inspiration, it passed with the air into the windpipe. There was evidence that this foreign body was still in or near one of the bronchi. The child is five years old, of excellent constitution and good health.

"The usual distressing symptoms followed the introduction of this extraneous substance into the air passages, and efforts were immediately taken for its expulsion. Emetics were given; the patient was held up by the heels, and stricken repeatedly between the shoulders and on the sternum, but these means failed.

"Drs. Ford, McKie, J. A. Eve, Henry and Robert Campbell, were the consulting physicians called into the case here. There was a large bronchial rhonchus, distinct at a distance, in both lungs, but chiefly in the left. There were also different degrees of sibilant rhonchus, alternating with the moist. This examination could not, however, be critically made, owing to the extreme repugnance of the patient and his consequent restlessness. The foreign body was not large enough to occlude one bronchus; and besides, the irritation created by it might readily have extended into both, in the space of two weeks, that interval having now elapsed since the accident occurred—so that the exact location of the nail could not thus be determined. There was a cough, with some expectoration, every morning, and occasional dyspnœa, especially after exercise. The disturbance to his general system was but slight; his appetite was good and he went about as a child without restraint.

"After watching the case for a few days, we came gradually, but unanimously to the conclusion, to recommend the operation of tracheotomy for the removal of the nail. It is proper to state that the possbility of acting upon it through the agency of magnetism was duly considered and experiments performed with this object, but leading to no available practical results. The forceps used in the case was magnetized, but exercised no perceptible influence in the extraction of the foreign body.

"On the 27th, kindly and efficiently aided by the professional gentlemen above named, and by Prof. Means, who administered chloroform, Drs. Broadhurst, Dearing, and Simmons, the operation was performed as follows:—The patient having the neck made prominent by a pillow under it, the integuments were raised in a fold over the *trachea* and divided from within outwards to about two and a half inches in length. The dissection was then cautiously continued upon the median line with the handle of the knife, forceps, and director; passing the platisma myoides (not recognized, however,) cellular tissue and adipose matter, between the sterno-hyoid and sterno-thyroid muscles, and opening the trachæl fascia described by Porter, of Dublin, the windpipe was exposed to about its fifth cartilaginous ring. At this point of the operation the two middle thyroid veins, running directly from the thyroid body or gland, as it is commonly called, into the venæ innomenatæ were greatly exposed, and at every struggle of the patient or difficult respiration, would become largely distended. By means of blunt hooks all the soft parts were carefully held aside, and no important vessel was injured. The whole bleeding was probably less than an ounce, and proceeded from the first or superficial incision. It was quite a bloodless operation, considering the region involved, and may be attributed to acting rigidly upon the principles to keep directly upon the median line; to have the cutting edge of the knife always turned upward; and to be chary even with its point in laying bare the trachea. Thus fully exposed, a hook secured the windpipe, while some three or four of its rings were rapidly slit open with a small knife, turning its back as much as possible toward the vertebræ. Trousseau's tracheal forceps were now introduced, and between its expanded blades other curved forceps were passed down into the right bronchus. These latter instruments were made in this city by Mr. J. D. Smith, are of different lengths, varying from three to seven inches in their narrow legs, and have considerable curvature near the handles or rings. Each introduction of them was attended with slight spasm, but which the subsequent manipulation, (gentle, of course,) in the bronchial tubes did not increase. Closed and used as a probe, these forceps were carried into the right bronchus, but their handles coming in contact with Trousseau's instrument holding open the wound, I could not determine if the nail had been touched. Calling for a probe, Dr. H. Campbell passed it down and readily detected it on the left side, where it was at once seized and extracted with the forceps. From my position to the right of the patient, and the hand sustaining his head being placed under the right side of his chin, the right bronchus was easiest penetrated. To find the object searched for, I had to request the assistant holding the patient's head, to withdraw his hand, while I passed my right hand carrying the forceps to the right of the neck. I am thus particular to prove that the nail must have been in the *left* bronchus and not in the right, as is almost invariably the case with extraneous substances passing down the windpipe, especially if they be, like this one, ponderous. The body removed could not have been more than two inches below the top of the sternum; was at an angle with the perpendicular line, and situated more anteriorly than I expected to find it. Its head was downward, and is very rough. It measures nearly an inch and a half in length and is slightly oxydized.

"The patient was about half an hour on the table, some portion of which time was consumed in waiting for all bleeding to cease before the trachea was opened, and also by his vomiting freely, the stomach having been filled by misplaced kindness to prepare him for the operation, and contrary to all expectation. For success in the case, I am greatly indebted to *chloroform*, which was admirably regulated to an extent sufficient for all purposes, yet never once producing stertorous breathing — to Dr. Ford for insisting that the extracting forceps should be curved greater than I intended — to Dr. Henry Campbell, for so readily touching the nail with a probe — and to Trousseau's tracheal forceps for keeping the parts dilated when cut open. Climate and season too, no doubt had their influence over the happy result. The operation was performed in an open piazza, with the thermometer at 48°.

"The after treatment of the case was of the simplest character. Two sutures applied to the skin had to be removed on account of threatened emphysema, and all dressing to the wound omitted, owing to the alarm of the patient recovering from chloroform. Two hours after the operation, he was taken in a carriage to his boarding-house, distant from mine two and a half squares. During the after part of the day, bloody mucus was still discharged with air through the tracheal factitious opening whenever he fretted or cried; but when composed he breathed altogether *per vias naturales*. At 8 o'clock, P. M., he was sleeping quietly and had less mucous rattle.

"*June* 28*th*, 6 *o'clock*. — Has had a good night. Has very little fever. The wound was partially closed at 11, and effectually at 7, P. M., with isinglass plaster. The lungs are much freer, and the respiration improved. The diet, up to this period, has been ice-lemonade, and milk and sugar, the latter his favorite and accustomed nourishment.

"29*th*, 7, *A. M.*— Is doing well; but at 11 has considerable fever. Prescribed calcined with the sulphate of magnesia. This being vomited, salt water enemata were directed. 8, P. M., the fever has abated, but the prescriptions have not been carried out, and there has been no action yet in the bowels. The wound remains closed, and his cough has ceased.

"30*th*.— Patient is down stairs and it is difficult to keep him in doors. His bowels have been moved twice. The wound was dressed, found healed internally leaving the skin ununited.

"*July* 1*st*.—Has left by railroad for the country.

"2*d*.—Came in to have the wound dressed — it is nearly healed, but the cicatrix is a little irregular.

"4*th*.— Left for home.

"*Recapitulation of case*.— Patient aged 5 years. Gets a fourpenny or shingle-nail into the left bronchus, from which it is removed through an opening in the trachea by forceps, three weeks after its sojourn in the air-passages. Within four days after the operation, he goes into the country and fully recovers, for he has been heard from several times after the above date."

